# *Capsicum* Endophytic Bacterial Strain LY7 and Prochloraz Synergistically Control Chilli Anthracnose

**DOI:** 10.3390/jof10030169

**Published:** 2024-02-22

**Authors:** Lu Ren, Nan Qin, Junqi Ning, Hui Yin, Hong Lü, Xiaojun Zhao

**Affiliations:** 1 College of Plant Protection, Shanxi Agricultural University, Taiyuan 030031, China; renlubaby@163.com (L.R.);; 2 Key Laboratory of Sustainable Dryland Agriculture (Co-Construction by Ministry and Province), Ministry of Agriculture and Rural Affairs, Taiyuan 030031, China

**Keywords:** capsicum anthracnose, biological control, *Bacillus*, bacterial–fungicidal combination

## Abstract

Chilli anthracnose is a major infectious disease of the genus *Capsicum*. Chemical control is the primary means of controlling this disease; however, the excessive use of chemical pesticides can adversely affect ecological security and human health. Here, our aim was to explore the synergistic effects of chemical and biological pesticides in the control of chilli anthracnose. The bacterial strain LY7, which is antagonistic to the anthracnose-causing fungus *Colletotrichum scovillei*, inhibited the growth of *C. scovillei* by 83.52%. Through morphological and genetic analyses, this strain was identified as *Bacillus velezensis*. Then, the compatibility of LY7 with three common chemical fungicides was determined. The in vitro protective and therapeutic efficacies of the 1 × 10^9^ CFU/mL (colony-forming unit/mL) bacterial solution were 66.38% and 35.18%, respectively, but both were significantly lower than those of prochloraz, the most compatible fungicide. We then conducted field efficacy trials to elucidate the best combination of prochloraz and LY7; the highest control efficiency was achieved with a suspension of 1.0 × 10^8^ CFU/mL of LY7 mixed with 0.75 g/L prochloraz (3:7 ratio). Electron microscopy revealed the inhibitory effects of LY7 and prochloraz on *C. scovillei* mycelial growth. These results suggest that an LY7-based biofungicide can partially replace prochloraz, serving as an integrated management strategy to control chilli anthracnose.

## 1. Introduction

*Colletotrichum* fungi (*Colletotrichum* spp.) are a class of pathogens that infect various plants and cause destructive diseases. Over 167 *Colletotrichum* species cause over 200 plant diseases worldwide [1,2]. Among these, capsicum anthracnose is caused by infection with at least four species (*C. gloeosporioides*, *C. acutatum*, *C. tuncatum*, and *C. coccodes*) and causes serious damage to capsicum fruits by not only reducing their yield but also by reducing their marketability [2,3,4,5]. The pathogens of capsicum anthracnose vary by country and region. For example, *C. acutatum* is the main pathogenic species responsible for capsicum and sweet pepper anthracnose in South Korea and Taiwan, *C. truncatum* is dominant in Thailand and India, and *C. gloeosporioides* is the most common in Indonesia and most parts of China [6]. Consequently, capsicum anthracnose pathogens have different regional characteristics. A previous study found that the etiological agents of *Colletotrichum*-infected capsicum in Shanxi Province, China, include *C. gloeosporioides*, *C. scovillei*, *C. acutatum,* and other species; among these, *C. scovillei* occurs in different geographically isolated chilli producing areas and is the main pathogen responsible for the occurrence of capsicum anthracnose in Shanxi Province. According to Gao et al. [7], *C. scovillei* is also widely prevalent in Shandong Province, China. It is extremely destructive, causing a 40% decline in chilli yields, and can infect 42 types of cash crops, including apple, citrus, mango, and strawberry fruits.

Owing to a lack of high pesticide resistance in chilli varieties, fungicides such as aromatics, organic sulphur, benzimidazole (MBCs), methoxyacrylate fungicides (QoIs), and sterol demethylation inhibitors (SDIs) are important means of controlling this disease. However, the widespread use of fungicides has induced different degrees of resistance in *C. gloeosporioides* and *C. acutatum* to single-site fungicides such as MBCs and QoIs [8,9].

To reduce the adverse effects of chemical pesticides, considerable effort has been expended to identify biocontrol microorganisms for chilli anthracnose control. Various types of biocontrol organisms have been identified for capsicum anthracnose, including antagonistic bacteria, fungi, and actinomycetes. Among antagonistic bacteria, *Bacillus* has the advantages of a broad antibacterial spectrum, fast reproduction, high stress resistance, stable disease-controlling effects, high biological security, and good plant growth promotion; thus, this genus holds great potential for further research. Mi et al. (1993) proposed that the action mechanism of a compound biocontrol agent may benefit from different control mechanisms and variable effects at different sites in the plant body or soil to reduce competition and inhibit pathogenic bacteria through the secondary metabolites produced [10].

Biopesticides can improve crop yields while offering the advantages of being pollution-free, economical, and effective; these agents have facilitated remarkable achievements in the prevention and control of plant diseases. However, numerous problems, such as the instability of the biocontrol effects in practical application and the development of corresponding corrective measures, remain to be solved regarding their use. Furthermore, the long-term irrational use of chemical pesticides is damaging human and animal health, polluting soil and water, and considerably affecting the ecological balance [11]. The combined application of chemical pesticides and bacterial biocontrol agents is an effective way to control plant diseases. Combining biocontrol bacteria and low-dose chemical pesticides for plant disease control can not only improve the stability of biocontrol bacteria but also substantially reduce the number of chemical fungicides required, thus diminishing the chances of pesticide resistance development, environmental pollution, and other related problems. Anand et al. [12] found that the use of *Pseudomonas fluorescens* Pf1 in combination with 50% reduced pyrimethanil was as effective in controlling capsicum anthracnose as standard fungicides.

Regarding the synergistic effects of bacteria and fungicides, *Bacillus* is a common biocontrol bacterium that is increasingly used in combination with different pesticides for prevention [13]. Currently, the main *Bacillus* taxa used for biological control include *B. subtilis*, *B. amyloliquefaciens*, *Paenibacillus polymyza*, *B. methyltrophic*, and *B. velezensis* [14]. Peng et al. [15] found that the highly effective biocontrol agent *B. subtilis* B-001, when used in combination with Saisentong in root irrigation or as a spray, provided better disease prevention effects than either agent alone.

In this study, our objective was to explore the synergistic disease-control effects of chemical and biological pesticides against chilli anthracnose to provide a reference for reducing the required dosage of chemical pesticides and improving the stability of biological pesticides. To this end, we screened and taxonomically identified antagonistic bacteria with inhibitory effects against capsicum anthracnose; screened chemical fungicides used to prevent and control capsicum anthracnose for good compatibility with biocontrol bacteria; and performed a field efficacy test of different combinations of bacteria and fungicides for capsicum anthracnose control. This study will provide a theoretical basis for controlling chilli anthracnose.

## 2. Materials and Methods

### 2.1. Endophytic Bacterial Strains and Plant Material

The endophytic bacterium ZJ1 was isolated from the stems of *Buddleja lindleyana*; LMS1, LMS3, and LMS5 were isolated from the spikes of *Chenopodium quinoa*; YPTJ2 and YPTJ3 were isolated from the stems of *Vitisbryoniifolia bunge*; and HLJ5 was isolated from the stems of *Chenopodium quinoa*. The endophytic bacteria LY7 and LY8 were isolated from the leaves of *Capsicum annuum* L. and LJ6 from the stems of *Capsicum annuum*. The pepper-derived endophytic bacteria were isolated in this study, whereas those from other plants were previously isolated in the laboratory. The previously isolated bacteria were verified to exhibit a broad spectrum of antifungal effects.

The isolation method was based on that described by Wang et al. [16]. Fresh pepper tissues (roots, stems, leaves, and fruits) were washed with running water for 2 min and then left until dry. The tissue was sterilized with 75% ethanol solution and 3% sodium hypochlorite solution on the bechtop and washed with sterile water 3–5 times. The sterile water-coated plate of the last rinse was used as a blank control. Pepper leaves and fruits were cut into pieces (5 × 5 mm) with a sterile knife, and roots and stems were fully ground with a sterile mortar. The tissues were then inoculated on the surface of nutrient agar (NA; each 1 L contained 10 g of peptone, 5 g NaCl, 3 g beef extract, and 15 g agar; pH 7.4–7.6) in solid medium and incubated at 28 °C for 2−3 days and at 37 °C for 24 ± 2 h. After colony growth, single colonies were selected and inoculated into a new NA solid medium by the stripe separation method. After purification and separation for at least three generations, the obtained pure strains were stored at 4 °C for later use. Endophytic bacteria were originally isolated from single colonies grown in nutrient agar (NA) medium (each 1 L contained 10 g of peptone, 5 g NaCl, 3 g beef extract, and 15 g agar; pH 7.4–7.6) for 48 h at 28 °C. All endophytic bacteria were cultured on the NA medium at 28 °C for up to 3 d before use.

The *Colletotrichum scovillei* pathogen was isolated from diseased plant samples obtained from Shanxi Province, China. The isolates were cultured on potato dextrose agar (PDA; each 1 L contained 200 g potato, 20 g lactose, and 15 g agar; pH neutral) plates and incubated at 25 °C for up to 5 d before use. All isolates were stored at 4 °C.

Chilli plants (variety: Zhongjiao No. 108) were grown and provided by Zhongshu Seed Industry Technology Co., Ltd., Jinzhong, China and chilli seedlings (variety: Jianjiao No. 22) were grown and supplied by Zhongyu Seed Industry Company, Jinzhong, China.

### 2.2. Chemical and Biological Reagents

Chemical fungicides were commercially available. The fungicides 97.30% tebuconazole, 97.22% prochloraz, and 97.80% pyraclostrobin were purchased from Shandong Weifang Runfeng Chemical (Weifang, China) Co., Ltd., and 450 g/L prochloraz was purchased from Andao Maihuifeng (Yancheng, China) Co., Ltd.

The LY7 suspension was formulated as follows: 10% LY7 strain powder, 0.06% preservative methylparaben, 2% wetting dispersant sodium methylene bisnaphthalene sulfonate, 1.5% calcium lignosulfonate, 1.5% Tween-80, 0.3% thickening agent sodium carboxymethyl cellulose, 4% antifreeze glycerol, and 0.3% UV (ultraviolet) protection agent sorbitol were dissolved in distilled water.

A fermentation liquid of the LY7 strain, pre-frozen at −80 °C, was processed into raw powder via vacuum freeze-drying (SCIENTZ-12N freeze-dryer, Ningbo Xinzhi Biotechnology Co., Ltd., Ningbo, China). The raw powder was mixed with other additives according to the provided formula and then supplemented with distilled water to prepare a 1.0 × 10^10^ CFU/mL cell suspension. The additives of the LY7 strain suspension agent were screened using a biocompatibility test and optimized through formulations.

### 2.3. Screening of Antagonistic Bacteria

A ring of bacterial colonies was selected from the NA medium, inoculated into 250 mL triangular bottles containing 100 mL Luria Broth (LB; each 1 L contained 10 g peptone, 5 g NaCl, and 3 g beef extract; pH 7.4–7.6), and incubated at 25 °C with constant shaking at 160 rpm for 12 h to prepare the seed liquid. The seed liquid, with an inoculum volume of 1%, was then transferred to the LB medium and incubated at 25 °C with constant shaking at 160 rpm for 36 h. The bacteria were harvested by centrifuging at 12,000 rpm for 10 min at 4 °C, following which they were suspended in sterile water, with the concentration of the solution adjusted to 1 × 10^8^ CFU/mL. This solution was stored at 4 °C for subsequent use.

Using the Oxford cup method [17], with the center of the dish used as the center point cross and vertical cross in the sterile Oxford cup at four points 30 mm away from the center, 10 mL of PDA medium was poured into the Oxford cup for solidification. Endophytic bacterial liquid (100 μL) was injected into the centers of four Oxford cups with 5 mm diameter phytopathogenic fungus spots. The cups were then placed in a 25 °C-constant temperature incubator. Phytopathogenic fungus alone was used as a control. When the diameter of the control pathogen colonies reached approximately 6 cm, the pathogen colony diameter and inhibitory zone under all treatments were measured using Vernier calipers. The inhibitory rate was calculated using Equation (1) as follows:

(1) $$Inhibitory\;rate\left(\%\right)=\frac{C_{d}-T_{d}}{C_{d}}\times 100$$

where *C_d_* is the fungal colony diameter on the control PDA base plate and *T_d_* is the fungal colony diameter on the treatment PDA base plate.

### 2.4. Compatibility of Antagonistic Bacteria and Fungicides

The dilution-coated plate method was used to test the compatibility of the screened antagonistic bacteria with different fungicides. The three fungicides with strong inhibitory effects against *C. scovillei* identified in the pretest (tebuconazole, prochloraz, and pyraclostrobine) were mixed with sterile water to obtain five concentrations: 0.13, 0.25, 0.5, 1, and 2 μg/mL for tebuconazole; 0.033, 0.065, 0.13, 0.25, and 0.5 μg/mL for prochloraz; and 0.02, 0.08, 0.1, 0.4, and 2 μg/mL for pyraclostrobine. These concentrations were determined based on the inhibition rate of each fungicide against *C. scovillei* in the pretest (the inhibition rates at the highest and lowest concentrations were approximately 85% and 15%, respectively). Then, 20 μL of each antagonistic bacterium was injected into the fungicide-containing medium, and the plates were uniformly coated using an aseptic coating rod. As a blank control, the fungicide was replaced with an equal volume of sterile water. At 3 d post inoculation, fungal colonies were counted, and the survival rate was calculated using Equation (1). The experiment was repeated independently at least three times for each concentration.

### 2.5. Identification of Strain LY7

The morphology of the bacterial colony of LY7 was observed and recorded after it had been subjected to gradient dilution with sterile water and incubated on an NA medium for 5 d at 25 °C. The seed liquid was transferred to the LB medium at a 1% inoculum volume, centrifuged at 160 rpm, and incubated at 25 °C for 24 h. This was followed by centrifugation at 4 °C and 10,000 rpm for 10 min to obtain the bacteria. Following the method of Jin et al. [18], the supernatant was discarded, and the precipitation was left in the pipe according to the volume ratio of 1:20. The fixative glutaraldehyde was added, mixed well, and then poured into a 2 mL centrifuge tube at 4 °C and 10,000 rpm for 10 min. The supernatant was removed, the bacteria were collected, and a PBS buffer with pH 7.4 and a concentration of 0.1 mol/L was added to shake and rinse twice for 10 min each time. The bacteria were evenly coated on the cover glass and dehydrated in a series of ethanol solutions at concentrations of 30%, 50%, 70%, 80%, 90%, and 100% for 10 min each, and finally dehydrated with tert-butanol for 10 min. After drying, the gold spraying was performed. Finally, the morphology and size of the LY7 bacteria were observed using scanning electron microscopy (SEM, JSM-6490LV, Nippon Electronics Co., Ltd., Tokyo, Japan).

Genomic DNA was extracted from the LY7 strain according to the bacterial DNA extraction method described by Hasan et al. [19], and this DNA was used as a PCR amplification template. The amplification primers used in this experiment were provided by Shengong Bioengineering (Shanghai, China) Co., Ltd. (Shanghai, China). Universal bacterial primers 27F (5′-AGAGTTTGATCCTGGCTCAG-3′) and 1492R (5′-GGTTACCTTGTTACGACTT-3′) [20] and gyrA-specific primers gyrAF (5′-CAGTCAGGAAATGCGTACGTCC-3′) and gyrAR (5′-CAAGGTAATGCTCCAGGCATTGCT-3′) were used as amplification primers [21]. The reaction mixture contained 12.5 μL Primermix, 2 μL primers, 1 μL DNA, and 9.5 μL double distilled water. The cycling conditions for 16S rRNA were as follows: pre-denaturation at 94 °C for 5 min, followed by 30 cycles of denaturation at 94 °C for 30 s, annealing at 57 °C for 30 s, extension at 72 °C for 90 s, and a final extension at 72 °C for 10 min. The cycling conditions for gyrA were as follows: Pre-denaturation at 94 °C for 5 min, followed by 30 cycles of denaturation at 94 °C for 45 s, annealing at 57 °C for 45 s, extension at 72 °C for 45 s, and a final extension at 72 °C for 10 min. The PCR amplification products were sequenced by Shengong Bioengineering (Shanghai, China). The sequences were compared and analyzed using the NCBI BLAST search engine (https://blast.ncbi.nlm.nih.gov/Blast.cgi, accessed on 20 March 2022). Related sequences with high similarity were selected, and a phylogenetic tree was constructed using MEGA version 7 (Mega Limited, Auckland, New Zealand) to clarify the classification status of strain LY7.

### 2.6. In Vitro Disease-Controlling Effects of Strain LY7

Bell peppers of the same size and maturity stage were cleaned with sterile water and disinfected with 75% alcohol three times. After their surfaces were dried, they were placed in sterilized disposable boxes. Fruit wounds were made through sterile inoculation, and 5-mm *C. scovillei* spots were prepared for wound inoculation. LY7 suspensions of 1 × 10^6^, 1 × 10^7^, 1 × 10^8^, and 1 × 10^9^ CFU/mL were prepared, and 450 g/L of 600× diluted prochloraz and sterile water were used as the positive and blank controls, respectively.

First, the pepper fruits were sprayed with different treatment solutions until they were completely infiltrated; after 24 h, the fruits were inoculated with *C. scovillei* spots. In a second experimental group, *C. scovillei* spots were inoculated, and, after 24 h, the pepper fruits were sprayed with different treatment solutions until they were completely infiltrated. Three replicates were performed for each experiment, with five fruits in each replicate and three wounds applied to each fruit. The disease index was determined on day 7, and the disease-controlling effects were calculated according to Equations (2) and (3).

The grading criteria for the disease of pepper fruits were as follows: grade 0: no disease spot; grade 1: spot diameter ≤ 0.5 cm; grade 2: spot diameter 0.5–1.0 cm; grade 3: spot diameter 1.0–2.0 cm; grade 4: spot diameter 2.0–3.0 cm; grade 5: spot diameter > 3.0 cm.

Disease index = [(Σ (number of spots at all levels × relative progression)/(total number of spots investigated × 5)] × 100 (2)

Prevention and treatment effect % = (disease index of control group − disease index of treatment group)/disease index of control group × 100 (3)

### 2.7. Effects of Combined Treatment with Strain LY7 and Prochloraz on C. scovillei Mycelial Growth

First, culture plates were prepared for each treatment. The following treatments were applied: Treatment 1 comprised LY7 strain suspension at the effective concentration for 50% inhibition of mycelial growth dose (EC_50_ dose), treatment 2 comprised prochloraz at the EC_50_ dose, and treatment 3 comprised an optimal proportion of LY7 suspension and prochloraz concentration. PDA medium was used as the control. *C. scovillei* spots (5-mm diameter) were inoculated in the center of each treatment culture plate, and three to four sterilized cover slides were inserted obliquely around each spot. Each treatment was repeated three times. When the mycelia reached the cover glass, they were removed with tweezers, and mycelial morphology was observed using SEM. SEM samples were prepared as described in Section 2.5.

### 2.8. Determination of the Toxicity of the Combination of Prochloraz and Strain LY7 to C. scovillei In Vitro

Prochloraz was mixed with sterile water and then added to the PDA medium to obtain final concentrations of 0, 0.025, 0.05, 0.1, 0.25, and 0.5 μg/mL. Briefly, the seed liquid was obtained as described in Section 2.3, transferred to the LB medium at a volume of 1% inoculum, and the culture was shaken for 36 h. The bacteria were obtained by freezing, centrifugation, and precipitation in a fermentation solution and then suspended in sterile water. The bacterial concentration was then adjusted to 1 × 10^4^, 1 × 10^5^, 1 × 10^6^, 1 × 10^7^, and 1 × 10^8^ CFU/mL.

The inhibition rate obtained with each concentration of the bacterial suspension was calculated using Equation (1) and then converted to an inhibition probability value. A regression equation (*y* = a*x* + b) was applied, where *x* is the logarithm of the mass concentration and *y* is the probability value. The EC_50_ values and correlation coefficients (r) were calculated.

### 2.9. Optimisation of the LY7 Strain and Prochloraz Mixture

The Horsfall method [22] was used to evaluate the synergistic effects of different combinations of LY7 and prochloraz. Based on the prochloraz virulence test and strain LY7, the EC_50_ values of each solution were used to determine the working concentrations. The different volume ratios of prochloraz and strain LY7 (10:0, 9:1, 8:2, 7:3, 6:4, 5:5, 4:6, 3:7, 2:8, 1:9, and 0:10) were used to prepare a mixed solution. The same volume of sterile water equal to a mixed solution was used as a blank control; each experiment was performed in triplicate. When the control colony grew to approximately 6 cm, the diameters of the colony in the different treatment plates were measured, and the average inhibitory rate, the expected inhibitory growth rate, and the toxicity ratio were calculated (Equations (4) and (5)). The synergistic effects of the different formulations were determined based on the toxicity ratio, which was calculated as follows:

Toxicity ratio = actual inhibitory/expected inhibitory (4)

The expected inhibitory growth rate equation was calculated as follows:

Expected inhibitory = actual inhibitory rate of the LY7 suspension EC_50_ dose × percentage in the ratio + actual dose inhibitory rate of prochloraz EC_50_ dose × percentage in the ratio (5)

A toxicity ratio > 1 indicated a synergistic effect, a toxicity ratio < 1 indicated an antagonistic effect and a toxicity ratio = 1 indicated an additive effect.

### 2.10. Field Experiment

The field experiment was conducted in Yuci (Jinzhong, China) from 2021 to 2022 using chilli seedlings of the variety Jianjiao No. 22, which shows low resistance to *C. scovillei*. Cultivation and fertilization of the soil were performed according to appropriate agronomic guidelines. The width and height of the ridge in the experimental field were 0.5 m and 0.3 cm, respectively. The trials comprised eight treatments, each with four replicates. Each replicate covered an area of 30 m^2^. The soil in the test site was alkaline loam, with a pH value of 8.1 and medium organic matter content. The period from the first application to the end of the investigation was 25 days. The average temperature and relative humidity during the 2021 and 2022 tests were respectively 24.4 °C and 22.9 °C, and 79.96% and 77.34%.

The random squares method was applied. Treatments were as follows: (1) control (water), (2) 1.0 × 10^8^ CFU/mL LY7 suspension, (3) 5.0 × 10^7^ CFU/mL LY7 suspension, (4) 1.0 × 10^7^ CFU/mL LY7 suspension, (5) 0.75 g/L prochloraz, (6) 1.0 × 10^8^ CFU/mL LY7 plus 0.75 g/L prochloraz in a 3:7 ratio, (7) 5.0 × 10^7^ CFU/mL LY7 plus 0.75 g/L prochloraz in a 3:7 ratio, and (8) 1.0 × 10^7^ CFU/mL LY7 plus 0.75 g/L prochloraz in a 3:7 ratio.

In July and August of both years, at the beginning of the fruiting period when anthracnose occurs, the plants were twice sprayed with a handheld sprayer (1500 L/ha) at intervals of 7–10 d. No other fungicides were applied to the experimental plots.

The disease indexes were determined on day 7 after the last spray, and the control efficacy was calculated according to Equations (6) and (7). Grading of capsicum anthracnose was performed as follows: 0, no spots; 1, lesion area accounted for <2% of the fruit area; 3, lesion area accounted for 3–8% of the fruit area; 5, lesion area accounted for 9–15% of the fruit area; 7, lesion area accounted for 16–25% of the fruit area; 9, lesion area accounted for >25% of the fruit area.

Disease index = [(Σ (number of diseased fruits at all levels × relative progression)/(total number of fruits investigated × 9)] × 100 (6)

Control efficacy % = (disease index of the control group − disease index of the treatment group)/disease index of the control group × 100 (7)

### 2.11. Statistical Analysis

The EC_50_ values were obtained from a linear regression model, and 95% confidence intervals and correlation coefficients were calculated. Data regarding the antifungal effects of endophytic bacteria, effects of fungicides on LY7 strain colonies, efficacy of the combination of strain LY7 and prochloraz in controlling chilli anthracnose, and efficiency ratio underwent arcsine-square-root transformation before statistical analysis. Statistically significant differences were determined through analysis of variance, followed by Duncan’s multiple range test (α = 0.05). Data are presented as mean values ± standard error, and statistical significance was set at *p* < 0.05. All analyses were performed using the SPSS statistics software (version 20.0; IBM Corp., Armonk, NY, USA).

## 3. Results

### 3.1. Screening of Antagonistic Bacteria

**Figure 1:**
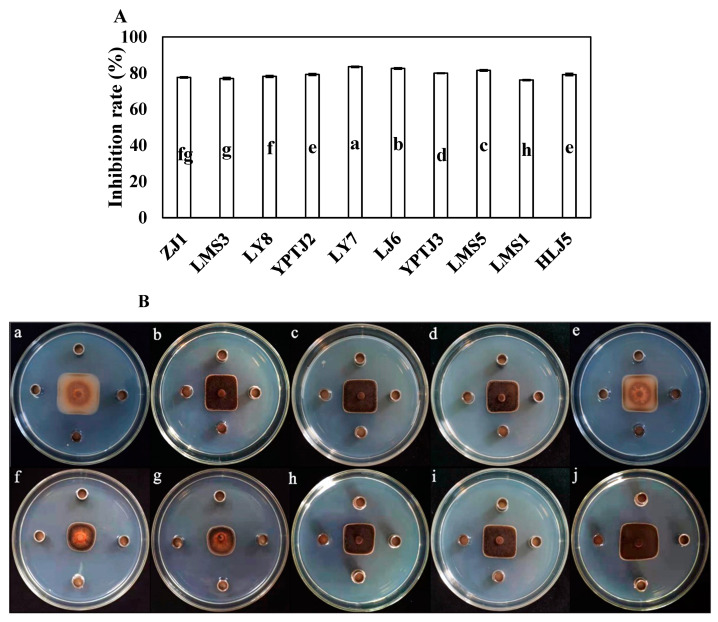
The antifungal effect of endophytic bacteria against *Colletotrichum scovillei.* (**A**) Different letters indicate significant differences per Duncan’s multiple range test (*p* < 0.05). (**Ba**–**Bj**) indicate the antifungal effect of strain ZJ1, LMS3, LY8, YPTJ2, YPTJ3, LY7, LJ6, LMS5, LMS1 and HLJ5 against *C. scovillei*.

The inhibitory rates of 10 endophytic bacteria against *C. scovillei* showed that strain LY7, isolated from healthy capsicum leaf, had strong antifungal effects against *C. scovillei* (Figure 1), with inhibitory rates of 83.52 ± 3.56%. The inhibitory rate of LY7 against *C. scovillei* was significantly higher than that against other endophytic bacteria (*p* < 0.05).

### 3.2. Compatibility of Antagonistic Bacteria and Fungicides

**Figure 2:**
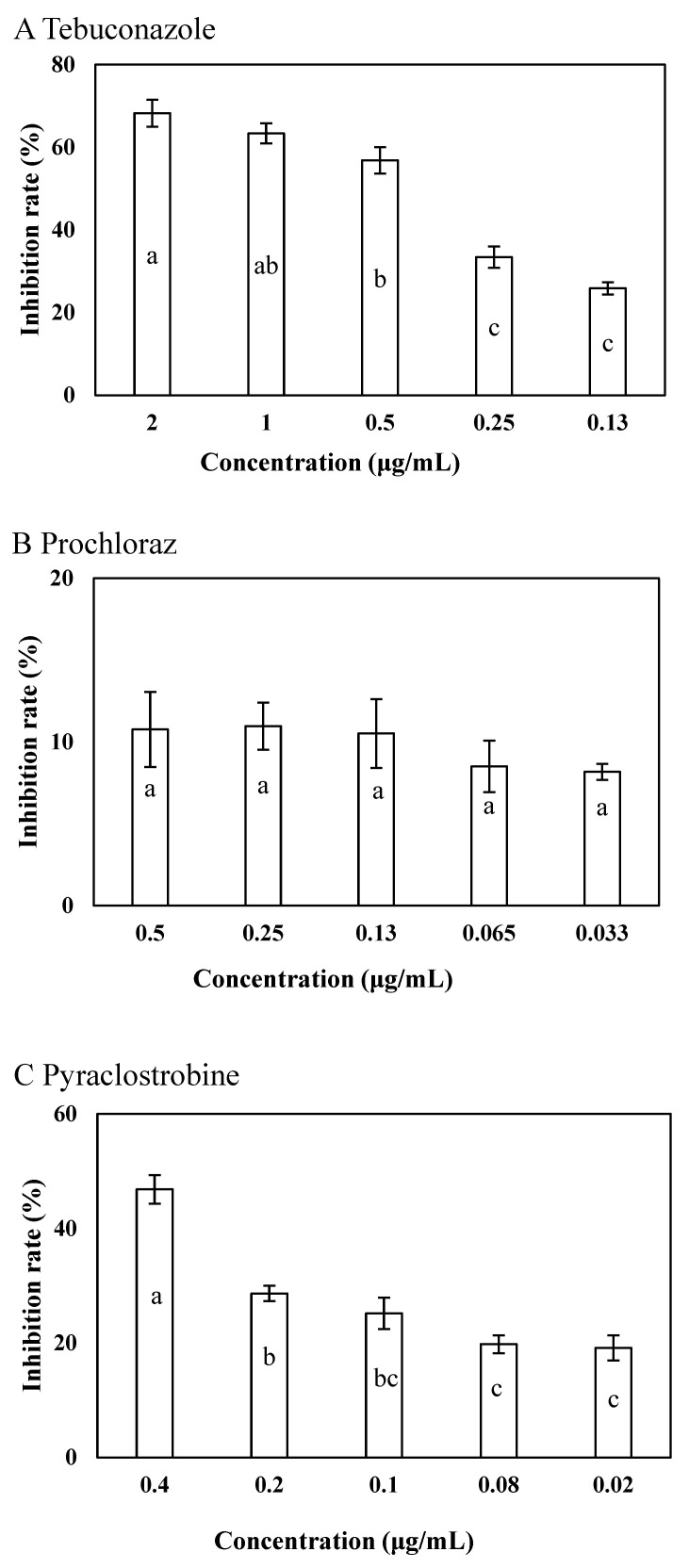
Effects of 3 fungicides on colony of LY7 strain and LJ6 strain. (**A**) The effect of tebuconazole against LY7 strain; (**B**) the effect of prochloraz against LY7 strain; (**C**) the effect of pyraclostrobine against LY7 strain. Different letters indicate significant differences per Duncan’s multiple range test (*p* < 0.05).

Tebuconazole significantly inhibited the growth of LY7 at a final concentration of 2 μg/mL, further exhibited an inhibition rate of 25.87 ± 3.41% at the 0.13 μg/mL concentration (*p* < 0.05) (Figure 2A). The LY7 strain demonstrated high compatibility with all the evaluated concentrations of prochloraz, with inhibitory rates of 8.18 ± 2.13% and 10.76 ± 2.29% at 0.033 and 0.5 μg/mL concentrations, respectively (*p* < 0.05) (Figure 2B). Pyraclostrobin also strongly inhibited the growth of LY7, with inhibitory rates of 46.82 ± 2.45% at 0.4 μg/mL and 19.13 ± 2.96% at 0.02 μg/mL concentrations (*p* < 0.05) (Figure 2C).

### 3.3. Identification of Strain LY7

**Figure 3:**
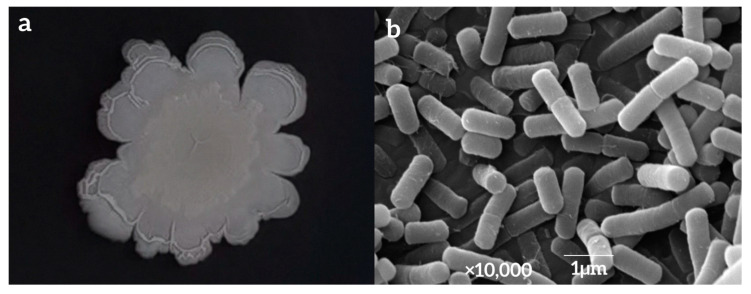
Photo of LY7 colony morphology (**a**) and strain LY7 by SEM (**b**).

The surface of the colony formed by strain LY7 in NA medium was dry and milky white, with a thick center and thin edges, a protrusion and folds in the center, irregular colony edges with surrounding folds, and no odor (Figure 3a). The bacterial bodies of strain LY7, as observed using SEM, were rod-shaped and periflagellar, with sizes of approximately 1.5–2.0 µm × 0.5–1.0 µm (Figure 3b).

**Figure 4:**
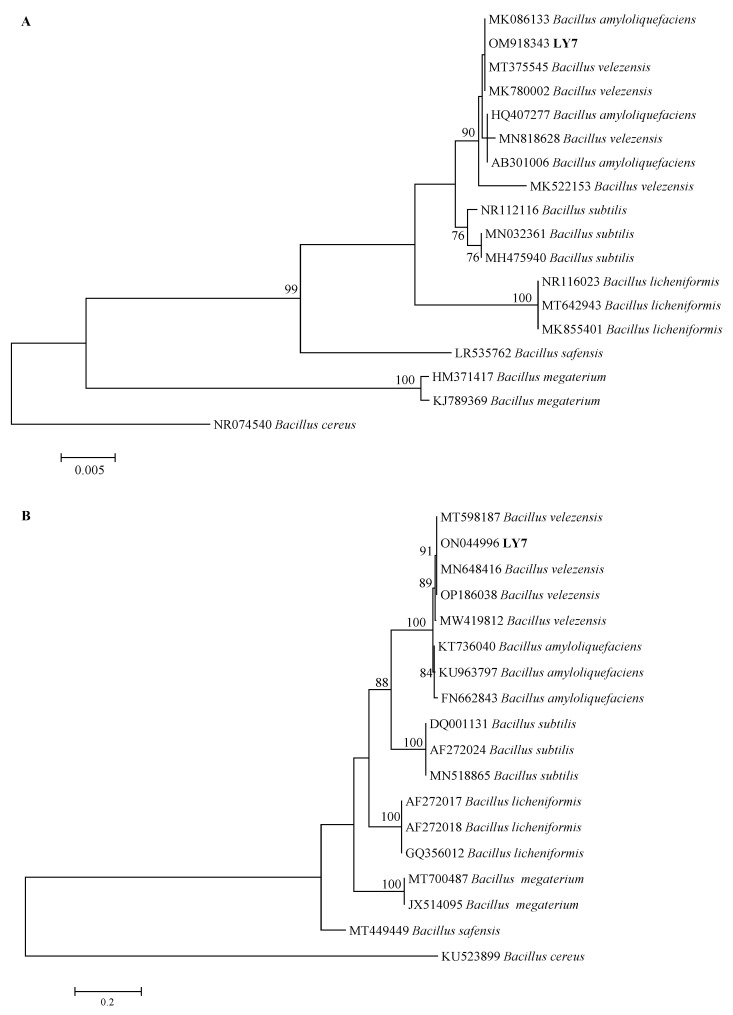
Phylogenetic trees for LY7 strain based on 16S rDNA and gyrA gene sequences. The percentage of replicate trees in which the associated taxa clustered together in the bootstrap test (1000 replicates) are shown next to the branches. (**A**) Neighbor-joining (NJ) tree showing the phylogenetic positions of the LY7 strain and other related taxa based on 16S rDNA gene sequences. *Bacillus cereus* was used as outgroup species. (**B**) Neighbor-joining (NJ) tree showing the phylogenetic positions of the LY7 strain and other related taxa based on gyrA gene sequences. *Bacillus cereus* was used as outgroup species.

LY7 16S rRNA and *gyrA* DNA were identified through PCR amplification and subsequent electrophoresis. After their quality was determined, the samples were sent to Shengong Bioengineering for sequencing. The 16S rRNA and *gyrA* sequence lengths were 1465 and 706 bp, respectively. The 16S rRNA and *gyrA* NCBI sequence accession numbers are OM918343 and ON044996, respectively. The *gyrA* sequence of the strain LY7 showed 100% homology with *Bacillus velezensis,* and it was therefore identified as *Bacillus velezensis* (Figure 4B).

### 3.4. In Vitro Disease-Controlling Effects of Strain LY7 against Capsicum Anthracnose

**Figure 5:**
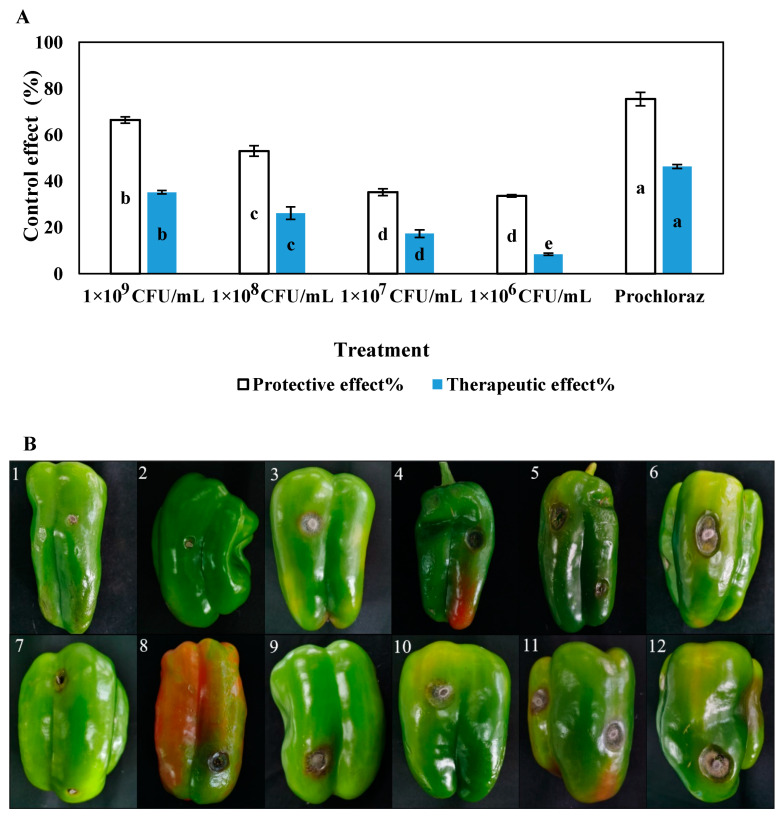
The control effect of different LY7 bacterial solution on *C. scovillei.* (**A**) Different letters indicate significant differences per Duncan’s multiple range test (*p* < 0.05). (**B1**–**B5**) indicate control effect of the protective effect followed by prochloraz, LY7 bacterial solution 1 × 10^9^ CFU/mL, 1 × 10^8^ CFU/mL, 1 × 10^7^ CFU/mL, 1 × 10^6^ CFU/mL, respectively; (**B6**) indicate control; (**B7**–**B11**) indicate control effect of the therapeutic effect followed by prochloraz, LY7 bacterial solution 1 × 10^9^ CFU/mL, 1 × 10^8^ CFU/mL, 1 × 10^7^ CFU/mL, 1 × 10^6^ CFU/mL, respectively; (**B12**) indicate control.

The in vitro disease-controlling effects of different concentrations of LY7 against chilli anthracnose differed significantly (*p* < 0.05) (Figure 5), with the protective effects being greater than the therapeutic effects at the same concentration. The protective and therapeutic effects of prochloraz were 75.42% and 46.31%, respectively, while those of the 1 × 10^9^ CFU/mL LY7 strain solution were 66.38% and 35.18%, respectively. The disease-controlling effects of the LY7 strain solution were significantly weaker than those of prochloraz. Furthermore, significant differences in the disease-controlling effects of different concentrations of LY7 were observed. With a reduction in bacterial count, the protective and therapeutic effects of LY7 also diminished. Those of the 1 × 10^6^ CFU/mL LY7 strain solution were 33.56% and 8.40%, respectively.

### 3.5. Effects of Combined Application of LY7 and Prochloraz on C. scovillei Mycelia

**Figure 6:**
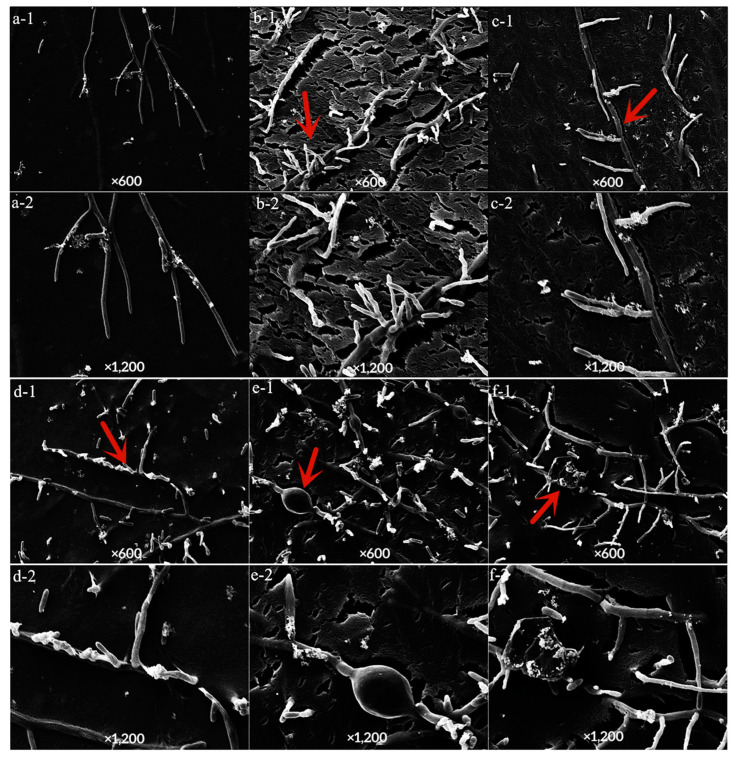
Effect of LY7 strain and prochloraz on the hyphae of *C. scovillei.* (**1,2**) indicate the hyphae map seen under 600 and 1200 time SEM, respectively; (**a**) indicate the hyphae of the control group, (**b**,**c**) indicate the hyphae of the LY7 strain mixed with prochloraz treatment; (**d**) indicate the hyphae of the prochloraz treatment; (**e**,**f**) indicate the hyphae of LY7 strain treatment. The red arrows in each subfigure were the observation positions.

The control mycelium showed divergent growth with a smooth surface (Figure 6a(1,2)), after treatment with LY7 and prochloraz alone, the mycelium showed uneven and distorted shapes (Figure 6d,f(1,2)). After LY7 treatment alone, the mycelium also showed multiple local expansions and fractures (Figure 6e,f(1,2)). Compared to in the absence of prochloraz treatment, the mycelium was more bifurcated and zigzagged, and the thickness was uneven with fractures after combined treatment with LY7 and prochloraz (Figure 6b,c(1,2)).

### 3.6. Optimal Mixing of LY7 and Prochloraz

The EC_50_ values of prochloraz and LY7 against *C. scovillei* were 0.135 and 3.1 × 10^3^ CFU/mL, respectively (Table 1). Table 2 shows a comparison of the synergistic effects of different proportions of strain LY7 and prochloraz. The 1.0 × 10^8^ CFU/mL LY7 suspension was mixed with prochloraz at a mixing ratio of 3:7, and the maximum toxicity ratio was 1.50 ± 0.04. The ratios of 2:8 and 6:4 (LY7:prochloraz) corresponded to toxicity ratios of 1.44 ± 0.03 and 1.42 ± 0.04, respectively, which were not significantly different from those associated with the 3:7 ratio (*p =* 0.06). Considering the inhibitory rate, the LY7 suspension mixed with prochloraz at a ratio of 3:7 was considered the optimum mixing ratio (Table 2).

### 3.7. Field-Control Efficacy Trial of LY7 in Combination with Prochloraz

**Figure 7:**
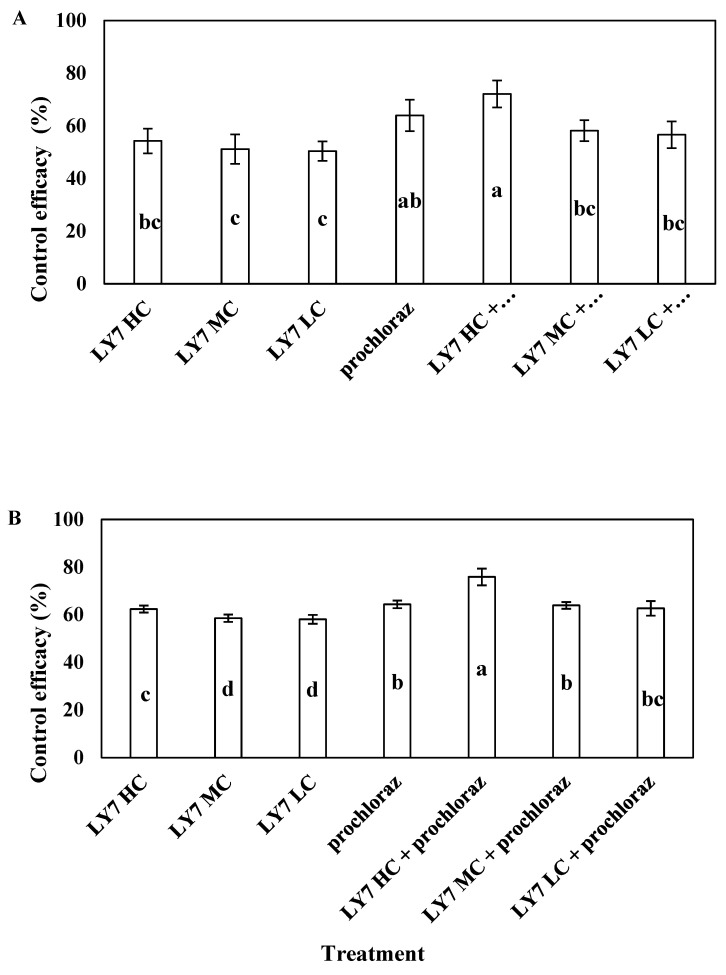
The control effect of LY7 suspension agent on chili anthracnos. (**A**) Indicate the control effect of 2021 year; (**B**) indicate the control effect of 2022 year. Different letters indicates significant differences per Duncan’s multiple range test (*p* < 0.05).

In the field trial, all treatments significantly reduced the severity of the disease. In 2021 and 2022, the incidence of anthracnose of pepper in the test site was high, with a disease index in the control of 19.17% and 23.64, respectively. In 2021, the control efficacy of 0.75 g/L prochloraz was 63.97%, whereas that of LY7 suspensions at 1.0 × 10^8^, 5.0 × 10^7^, and 1.0 × 10^7^ CFU/mL was 54.26 ± 4.70%, 51.16 ± 5.61%, and 50.39 ± 3.70%, respectively. There was no significant difference in the control efficacy of the three concentrations of LY7 suspensions used alone, but it was significantly lower than that of the LY7 suspension concentration at 1.0 × 10^8^ CFU/mL combined with prochloraz and that of prochloraz alone. Regarding the combination treatments, the control efficacy of 1.0 × 10^8^ CFU/mL LY7 suspension plus 0.75 g/L prochloraz was 72.09 ± 5.15%, which was not significantly higher than that of prochloraz alone (*p* < 0.05). The control efficiencies of the 5.0 × 10^7^ and 1.0 × 10^7^ CFU/mL LY7 suspensions mixed with 0.75 g/L prochloraz were 58.14 ± 4.02% and 56.59 ± 5.06%, respectively, which were not significantly lower than those of prochloraz alone (*F*_6,28_ = 3.882, *p* = 0.009) (Figure 7A). Collectively, the disease-control efficiency of all concentrations of LY7 suspension alone was lower than that of prochloraz, whereas that of LY7 suspensions mixed with prochloraz did not differ significantly from the efficiency of prochloraz alone.

In 2022, the control efficacy of 1.0 × 10^8^ CFU/mL LY7 suspension was significantly higher than that of 5.0 × 10^7^ and 1.0 × 10^7^ CFU/mL LY7 suspensions, and the control efficacy of the 1.0 × 10^8^ CFU/mL concentration of LY7 suspension mixed with 0.75 g/L prochloraz reached 75.82 ± 3.51%, which was significantly higher than that of prochloraz alone (64.28 ± 1.60%). These results indicate that prochloraz, combined with high concentrations of *B. velezensis* LY7, acted synergistically and could effectively control chilli anthracnose (Figure 7B). The effectiveness of the LY7 suspension plus prochloraz improved over two years of use.

## 4. Discussion

Chilli anthracnose is an important disease that affects pepper yield and quality. Chemical application is the most common method for controlling chilli anthracnose. The aim of this study was to screen out the antagonistic bacteria that can inhibit the anthracnose of capsicum and evaluate their inhibitory ability, thus providing at least a partial substitute for chemical fungicides.

Using the growth rate method, a preliminary test of the in vitro toxicity of eight fungicides revealed that tebuconazole, prochloraz, and pyraclostrobine inhibited mycelial growth to a greater extent than the other fungicides. However, the dependence on a single fungicide can cause resistance to plant disease and make disease control difficult [8]. In addition, the excessive use of chemicals leads to environmental pollution and threatens food security [23]. Therefore, biological control using antagonistic microorganisms has been studied extensively over the past few years. Endophytic bacteria that live in plant tissues and co-exist harmoniously with their hosts can protect plants by producing antifungal substances [24,25] without adverse effects on their host plants. However, the stability of the effects with biological control is relatively poor in production and practice; therefore, in this study, a combination of biological control with chemical control was evaluated.

In vitro bioassays revealed that some bacteria showed significant antifungal activity against *C. scovillei*. Ten strains of endophytic bacteria were screened for inhibitory effects on *C. scovillei*. Strain LY7 exhibited favorable antifungal effects, with an inhibitory rate of 83.52% and an inhibition zone of 12.75 mm. This strain was isolated from healthy capsicum plants. To efficiently control plant pathogens, the biocontrol agents applied to the leaves must successfully colonize the plant phyllosphere [26,27,28]. Biocontrol bacteria that subsequently enter the plant produce bioactive molecules that trigger the immune defense systems of the host plant or directly inhibit the development of the pathogen. Previous studies have shown that the LY7 strain can produce antifungal proteins and lipids; however, more research is needed to determine whether this bacterial strain can enter the plant through the leaves to colonize the organism.

The field experience of disease control shows that the application of most current biocontrol bacteria agents alone could not completely control the serious occurrence of plant diseases fashionable [29]. However, the use of biocontrol agents, either in a preventive role or used alternately or in combination with chemical agents, is inevitable in an environment where chemical agents exist. We tested the compatibility of three fungicides and the LY7 strain, and the growth of LY7 was mostly unaffected (8.18% inhibition) by 0.033 μg/mL prochloraz. Inhibition of 0.033 μg/mL prochloraz in LY7 was only 8.18%, leading us to conclude that there was sufficient compatibility for this pairing. Synergistic bacterial-fungicide control not only improves the stability of biocontrol bacteria in field applications but also reduces the required dosage of chemical fungicides. Due to the specificity of fungicides, most tend to be ineffective against bacteria, offering a greater possibility of compatibility for combinations of biocontrol bacteria, and various fungal fungicides have a greater possibility of compatibility. Studies have shown that the use of low-dose fungicides can inhibit pathogenic bacteria to a certain extent and help biocontrol bacteria stabilize colonization and become a dominant strain [30]. *Bacillus* has a strong tolerance to a variety of external harmful factors and a broad spectrum of antibacterial abilities, shows a wide distribution space, and most species are harmless to humans. Many studies have investigated the combination of *Bacillus* and fungicides. Zhang et al. (2020) found that the combination of 1 × 10^7^ CFU/mL GJ-22, which has a significant inhibitory effect on *Phytophthora infestans* in potatoes, and 700 mg/L cymoxanil had better disease-controlling effects than either single agent in field trials [31]. Anand et al. [12] showed that the inhibitory effect of Pf1 on *Colletotrichum capsici* was less pronounced than that of the standard-dose fungicide alone, but the combination of Pf1 and a 50% standard fungicide dose was as effective as the standard-dose fungicide alone. These findings indicate that such combinations can be successful in the prevention and control of a variety of plant diseases, and related application prospects have been expanded.

Morphological identification and 16S rDNA and *gyrA* gene sequencing were used to determine the taxonomic placement of strain LY7, which was identified as *Bacillus velezensis*. Owing to its rapid growth and strong antifungal activity, this strain shows great therapeutic potential. Some studies have demonstrated the utility of *B. velezensis* in biocontrol against various plant diseases [32]. Usually, the concentration ratio range of bacterial strains with substantial control effect is determined by in vitro culture tests as the basis of pot tests and field tests. In this study, the virulence of LY7 was determined using the growth rate method; the EC_50_ value of this strain was determined to be 3.1 × 10^3^ CFU/mL. The LY7 strain showed a significantly higher rate of inhibition of *C. scovillei* growth than the other tested strains. The toxicity ratio was 1.50 with the LY7 to prochloraz ratio of 3:7, indicating a synergistic effect probably resulting from the fungicidal mechanisms of both components.

The mechanism of action shown in vitro by LY7 appeared to be primarily driven by bioactive extracellular secondary metabolites. Metabolites exhibited antifungal properties in vitro and could be involved in the biocontrol effects observed in in vivo bioassays. A variety of lysogens and antifungal substances are usually present in the metabolites of biocontrol *Bacillus*, including ribosom-synthesized antifungal proteins (chitinase, glucanase, bacteriocin, etc.) and non-ribosom-synthesized antibiotics (including ictilin, phensubtilin, and subtilin liptide). According to Maachia et al. [33], *B. subtilis* can produce β-1,3 glucanase and chitinase, which participate in the dissolution of pathogenic fungi. The antagonistic effect of the LY7 strain on *C. scovillei* was observed in the present and resulted in mycelium digestion, bubble formation, destruction of growth points, and possibly the spilling of cell contents. Edwards et al. [34] suggested that *Bacillus* mainly inhibits the occurrence of plant diseases by secreting gramicitracin S, which can effectively kill spores, but has no significant inhibition effects on the growth of mycelium. However, the results of the present study showed that the LY7 strain could strongly inhibit the growth of mycelia. Demethylase inhibitors restrict mycelial growth more strongly than spore germination [35], as spores can reserve enough sterols to complete germination [36]. Therefore, to further clarify the antifungal mechanism of LY7, the effect of LY7 on the growth of *C. scovillei* mycelia was observed using SEM. The results followed those of previous studies. For example, Jin et al. [18] showed that *Aspergillus* inhibits mycelial growth and secretes lysozymes to degrade the cell walls of pathogens. This finding was confirmed by the antifungal activity of LY7 demonstrated in this study. We found that chemical fungicides improved some antagonistic substances and the bacteriolytic ability of biocontrol bacteria. The combination of chemical fungicides with different action mechanisms and *B. velezensis* directly affects the biocontrol function of *B. velezensis*. However, the mechanism of the effect of prochloraz combined with the LY7 strain on *C. scovillei* mycelia remains to be further studied.

Published reports on combined chemical and biological control have shown that combining fungicides and biological control microorganisms can improve the stability of plant disease-control effects [15,37,38]. In this study, the LY7 strain was used to prepare a bacterial suspension with adjuvants. Field efficacy trials, performed in 2021 and 2022, to determine the efficacy of single and combined treatments with different concentrations of agents revealed that the efficacies of 1.0 × 10^8^, 5.0 × 10^7^, and 1.0 × 10^7^ CFU/mL LY7 suspensions were lower than those of prochloraz alone; however, the 1.0 × 10^8^ CFU/mL LY7 suspension mixed with prochloraz and applied in 2022 was significantly more effective than prochloraz alone. Previous reports have indicated that the protective effect of biocontrol bacteria exceeds their therapeutic effect because inoculation of bacteria with a strong colonization ability can contribute to disease control [39]. Premature cell count decay can be avoided by adding a protective agent to the LY7 suspension. Consequently, the biocontrol agents would then carry out the biocontrol of fungal phytopathogens by promoting plant growth and defense mechanisms, which could reduce the use of synthetic pesticides. The LY7 strain suspension can be recommended as a partial substitute for chemical fungicides in a pepper plant production setting. The effectiveness test was conducted in a relatively controlled environment, and further field trials are needed before large-scale applications can be considered. However, our results provide useful information on the use of bacteriological agents to control capsicum anthracnose.

Notably, the control efficacy of the same concentration of LY7 suspension and prochloraz combined was higher in 2022 than in 2021. This may have been due to two possible reasons. First, a certain amount of time is required for LY7 to colonize the phylloplane of plants and soil and to occupy the dominant niche; thus, the control efficacy of biological fungicides is generally slower than that of chemical fungicides. In the first year of application, the control efficacy of the 1.0 × 10^8^ CFU/mL LY7 suspension alone was not significantly higher than that of 5.0 × 10^7^ and 1.0 × 10^7^ CFU/mL concentrations, and the combination of high concentration LY7 suspension and prochloraz was not significantly more effective than prochloraz alone. By 2022, the efficacy of the high-concentration LY7 suspension alone was significantly higher than that of the other two concentrations, and the effectivity of the combination of high concentration LY7 suspension and prochloraz was also significantly higher than that of prochloraz alone. Secondly, in addition to the fungicide or biological agent itself, the field control effect is also affected by the combination of many other factors, such as annual differences in climate factors, crop growth, and other influences. Biological bactericides are often more susceptible to environmental impacts than chemical fungicides. The combination of biocontrol bacteria and fungicides can make up for this limitation and provide a practical and environmentally friendly control method. Further study of the control effect of biocontrol bacteria preparations in different farmland environments and their combination with fungicides is required in the future.

## 5. Conclusions

The endophytic bacterium LY7, isolated from pepper leaves, showed good inhibitory effects against *C. scovillei* and demonstrated compatibility with prochloraz, resulting in synergistic fungicidal effects in a bacteria-to-fungicide ratio of 3:7. The control efficacy of the 1.0 × 10^8^ CFU/mL LY7 suspension mixed with prochloraz was higher than that of prochloraz alone. These findings indicate that the LY7 strain is a promising biofungicide. The LY7 suspension, when used at the recommended concentration and in a solution with prochloraz, can be applied for the control of capsicum anthracnose in the field. Our findings provide a useful foundation for developing new methods to control this important plant disease.

## Figures and Tables

**Table 1:** Evaluation of laboratory toxicity of three fungicides against *C. scovillei*.

Fungicide/Bacterial Solution	Toxicity Regression Equation	EC_50_^1^ μg/mL	Coefficient Correlation	95% Confidence Interva
Prochloraz	y = 2.83x + 2.47	0.135	0.996	0.114~0.159
LY7 strain	y = 0.48x − 1.72	3.1 × 10^3^ CFU/mL	0.995	1.8 × 10^2^~1.5 × 10^4^

^1^ EC_50_ = effective concentration for 50% inhibition of mycelial growth.

**Table 2:** The co-toxicity of LY7 strain and Prochloraz to against *C. scovillei*.

V (LY7):V (Prochloraz)	Actual Inhibitory (%)	Expected Inhibitory (%)	Toxicity Ratio ^1^
0:10	49.30 ± 1.75	50.00	1.00 d
1:9	74.28 ± 3.46	54.59	1.36 ± 0.02 bc
2:8	77.87 ± 2.31	54.00	1.44 ± 0.03 ab
3:7	80.13 ± 3.68	53.41	1.50 ± 0.04 a
4:6	72.54 ± 4.47	52.83	1.37 ± 0.01 bc
5:5	71.38 ± 2.19	52.24	1.36 ± 0.05 bc
6:4	73.49 ± 1.42	51.65	1.42 ± 0.04 ab
7:3	69.92 ± 1.07	51.06	1.37 ± 0.02 bc
8:2	70.51 ± 3.34	50.47	1.39 ± 0.08 bc
9:1	64.35 ± 1.88	49.89	1.29 ± 0.03 c
10:0	55.18 ± 2.32	50.00	1.00 d

^1^ The data are expressed as the mean values with the SE; Different letters indicates significant differences per Duncan’s multiple range test (*p* < 0.05).

## Data Availability

Data are contained within the article.
